# 6-Gingerol, a Major Ingredient of Ginger Attenuates *Diethylnitrosamine*-Induced Liver Injury in Rats through the Modulation of Oxidative Stress and Anti-Inflammatory Activity

**DOI:** 10.1155/2021/6661937

**Published:** 2021-01-19

**Authors:** Mohammed A. Alsahli, Saleh A. Almatroodi, Ahmad Almatroudi, Amjad Ali Khan, Shehwaz Anwar, Abdulmajeed G. Almutary, Faris Alrumaihi, Arshad Husain Rahmani

**Affiliations:** ^1^Department of Medical Laboratories, College of Applied Medical Sciences, Qassim University, Buraydah, Saudi Arabia; ^2^Department of Basic Health Sciences, College of Applied Medical Sciences, Qassim University, Buraydah, Saudi Arabia; ^3^Department of Medical Biotechnology, College of Applied Medical Sciences, Qassim University, Buraydah, Saudi Arabia

## Abstract

Diethylnitrosamine (DEN) is a well-known hepatocarcinogen, and its oral administration causes severe liver damage including cancer. DEN induces the pathogenesis of the liver through reactive oxygen species mediated inflammation and modulation of various biological activities. 6-Gingerol, a major component of ginger, is reported to prevent liver diseases by reducing the oxidative stress and proinflammatory mediators. The present study investigated the hepatoprotective effects of 6-gingerol through the measurement of oxidative stress, anti-inflammatory markers, liver function enzyme parameter, and histopathological analysis. The rats were randomly divided into four groups as the control, DEN treated (50 mg/kg b.w.), DEN+6-gingerol (each 50 mg/kg b.w.), and 6-gingerol only. To evaluate the hepatoprotective effects, liver function enzymes (ALT, AST, and ALP), oxidative stress markers (SOD, GSH, GST, and TAC), lipid peroxidation, inflammatory markers (CRP, TNF-*α*, IL-6, and ICAM1), haematoxylin and eosin staining, Sirius red staining, immunohistochemistry, and electron microscopy were performed. The results showed a significant increase in liver function enzymes, oxidative stress, and inflammatory markers in the DEN-treated group as compared to the control group. Besides this, altered architecture of hepatocytes (infiltration of inflammatory cells, congestion, blood vessel dilation, and edema), abundant collagen fiber and organelle structures like distorted shaped and swollen mitochondria, and broken endoplasmic reticulum were noticed. The administration of 6-gingerol significantly ameliorated the biochemical and histopathological changes. The increased expression of TNF-*α* protein was noticed in the DEN-treated group whereas the administration of 6-gingerol significantly decreased the expression of this protein. Based on these findings, it can be suggested that 6-gingerol may be an alternative therapy for the prevention and treatment of liver diseases.

## 1. Introduction

The liver is a main homeostatic organ, involved in several biochemical processes such as the metabolism of lipids, carbohydrates, and alcohol and detoxification of various toxins and metabolic waste products [1]. Continuous exposure of the liver to some factors including fatty diet, alcohol, drugs, and certain infections like virus leads to liver damage or injury. Sustained liver damage has been reported to cause chronic liver diseases [2]. Liver diseases are an important and certainly underestimated public health problem worldwide and are known to cause a substantial level of morbidity and mortality [3]. According to information on the global burden of disease project, more than two million people die from liver diseases that include acute hepatitis, cirrhosis, and liver cancer, which represent about 4% of all deaths around the world [4].

It is well-known that the liver is the centre for metabolism of various drugs and chemicals which makes it very susceptible to tissue damage by these compounds. Hence, these drugs and chemicals induce liver damage and alter the architecture of hepatocytes. Increased specific liver enzymes like alanine aminotransferase (ALT), alkaline phosphatase (ALP), and aspartate aminotransferase (AST) can be used as markers for the diagnosis of drugs and toxin-induced hepatotoxicity. Further, the discharge of mitochondrial enzymes from the liver might be a clear evidence of hepatic necrosis [5, 6] and is likewise connected with different liver diseases.

Besides, liver injury has been reported to be manifested by the generation of diverse free radicals and reactive oxygen species (ROS). Exposure to certain drugs and chemicals including DEN results in hepatocellular accumulation of ROS leading to oxidative damage of biomolecules including DNA [7]. In addition, inflammation has been known to be an important pathogenic event of liver injury, and ROS causes the induction of proinflammatory genes [8]. The excessive production of ROS [7] and overexpression of these proinflammatory genes play an important role in the severity of liver diseases [8].

Cytokines as inflammatory mediators are implicated in the pathogenesis of various acute as well as chronic diseases such as alcoholic liver disease [9–11]. It has been demonstrated that during chronic liver damage and activation of nuclear factor-*κ*-light-chain-enhancer of activated B cells, increased expression of TLR3 and TLR7 and increased secretion of proinflammatory cytokines are associated with human end-stage ALD [12].

DEN is an N-nitroso-alkyl compound and is a well-known hepatotoxin [13]. The parenteral or oral administration of DEN has been already observed to cause severe liver damage in experimental animals. DEN has been found in different food items such as cheese, soybean, cured meat, salted, and dried fish. In addition, DEN has been detected in tobacco smoke and baby bottle nipples. Humans are thus at potential risk of DEN exposure. Consequently, it becomes very necessary to search for effective and therapeutic medicines for hepatoprotection and management of liver diseases [14]. Antioxidants and anti-inflammatory agents have been recommended to be beneficial in the management of liver diseases [8]. Thus, the use of natural products that have the potential to protect from oxidative stress and inflammation can be considered as an important strategy in ameliorating hepatotoxicity.

Many herbs and their products have been used in the treatment of diseases due to their medicinal properties. Ginger or *Zingiber officinale* is a traditional dietary ingredient with notable medicinal properties like antioxidant, anti-inflammatory, antimicrobial, antihyperglycemic, antitumorigenic, and hepatoprotective activities [15]. 6*-*Gingerol is the chief pharmacologically active phenolic compound found in the rhizomes of ginger and has been demonstrated to exhibit a variety of biological activities such as antioxidant, anti-inflammatory, anticarcinogenic, and antimutagenic activities [16]. Furthermore, 6-gingerol has been reported to exert a therapeutic effect in osteoarthritis by means of attenuating oxidative stress as well as downregulation of proinflammatory mediators *in vitro* and *in vivo* [17]. Recently, 6-gingerol had been shown to induce cell death in the cancer cell line via caspase-3-dependent apoptosis and autophagy [18]. Further, 6-gingerol inhibits the metastasis of cancer cells and induces apoptosis in cancer cells [19, 20]. Henceforth, the present experimental study was accomplished to investigate the hepatotoxicity of DEN in rat liver and the protective ability of 6-gingerol against DEN-induced hepatotoxicity, oxidative stress, and inflammation.

## 2. Materials and Methods

### 2.1. Chemicals and Reagents

DEN and 6-gingerol were purchased from Sigma-Aldrich Chemicals Company (St. Louis, MO, USA). Assay kits including ELISA kits for inflammatory markers, antioxidant enzymes, and antibody TNF-alpha were purchased from Abcam, United Kingdom. The organic solvents (methanol, acetone, and ethanol), potassium dihydrogen phosphate (KH_2_PO_4_), ethylenediaminetetraacetic acid (EDTA), and hydrogen peroxide were of analytical grade.

### 2.2. *In Vitro* Study

#### 2.2.1. Evaluation of Antioxidant Activity of 6-Gingerol Extract by DPPH Assay

The diphenyl-2-picryl-hydrazyl (DPPH) assay was utilized to determine the free radical scavenging ability of 6-gingerol. Briefly, 10 mg/ml stock solution of the 6-gingerol was serially diluted in water to prepare a series of different concentrations (600-50 *μ*g/ml). Now, 1 ml of 0.3 mM DPPH working solution was prepared in absolute methanol and was mixed with 2.5 ml of various aqueous dilutions of 6-gingerol separately. The mixtures were kept in the dark for 30 minutes at room temperature. Then, the absorbance of various incubation mixtures was measured at 517 nm with a spectrophotometer against absolute methanol. To prepare the control, the 6-gingerol was replaced with methanol. All the experiments were run in triplicates. The percentage of free radical scavenging activity was calculated in line with the accompanying equation.

(1) $$\begin{array}{c} \%\text{ of free radical scavenging activity}=\left[\frac{A_{\text{control}}–A_{\text{sample}}}{A_{\text{control}}}\right]\times 100, \end{array}$$

where *A*_control_ is the absorbance of the control and *A*_sample_ is the absorbance in the presence of 6-gingerol.

#### 2.2.2. Reducing Power Estimation by Ferric Reducing Antioxidant Power (FRAP) Method

The ferric reducing antioxidant power (FRAP) method was conducted to evaluate the antioxidant potential of 6-gingerol with slight modifications [21]. In short, 1 ml of 6-gingerol (100 to 600 *μ*g/ml) in distilled water was added to different tubes having phosphate buffer (2.5 ml, 0.1 M, pH = 6.6). Now, potassium ferricyanide (2.5 ml, 1% *w*/*v*) was added to each tube. After proper mixing of solutions, the mixtures were incubated at 50°C for 20 min. At the end of incubation, 2.5 ml of trichloroacetic acid (10%) was included to each solution, and solutions were centrifuged at 3000 rpm for 10 min. Subsequently, 2.5 ml of the supernatant was collected and was mixed with 2.5 ml of distilled water. The resultant mixture was subjected to vortexing, and 500 *μ*l of ferric chloride (0.5%) solution (freshly prepared) was added to this. The absorbance of ascorbic acid (reference) and test samples was recorded at 700 nm against phosphate buffer (pH 6.6) as a blank solution. All the samples were run in triplicate. Higher absorbance belongs to higher reducing power.

#### 2.2.3. Hydrogen Peroxide (H_2_O_2_) Reducing Activity

The ability of 6-gingerol to reduce H_2_O_2_ was investigated to justify its antioxidant potential in accordance to the slightly modified method of Ruch et al. [22]. For this purpose, 40 mM solution of H_2_O_2_ was prepared in phosphate buffer (pH 7.4). 1 ml of H_2_O_2_ solution was added to different tubes having various concentrations of 6-gingerol (50-600 *μ*g/ml) in the absence of light. The solutions were mixed well using a vortex. The absorbance was recorded after 10 min using a UV-visible spectrophotometer at 230 nm. Phosphate buffer was used as blank solution. All the samples were run in triplicates. Percentage of H_2_O_2_ reducing activity was calculated using the following equation.

(2) $$\begin{array}{c} \text{Percentage of }{\text{H}}_{2}{\text{O}}_{2}\text{reducing ability}=\left[\frac{A_{\text{control}}–A_{\text{sample}}}{A_{\text{control}}}\right]\times 100, \end{array}$$

where *A*_control_ denotes the absorbance of the H_2_O_2_ solution and *A*_sample_ is the absorbance of the extract and H_2_O_2_ solution.

#### 2.2.4. Inhibition of Albumin Denaturation

Ginger has been reported to possess significant anti-inflammatory activity [23]. 6-Gingerol is a chief component of ginger. Therefore, the anti-inflammatory activity of 6-gingerol was investigated by using inhibition of albumin denaturation [24] using ibuprofen as a standard reference drug. In short, 500 *μ*l of 1% aqueous solution of bovine serum albumin (BSA) was added to Eppendorf tubes having 100 *μ*l of various dilutions of 6-gingerol (100-600 *μ*g/ml) or standard ibuprofen (200 *μ*g/ml). The pH of the solutions was adjusted to 7.4, and the tubes were first incubated at room temperature for 20 min. After the completion of 20 min, the tubes were heated at 71°C for 30 min. The reaction mixtures were left to cool down, and the turbidity of the reaction samples was determined by measuring absorbance spectrophotometrically at 660 nm against distilled water as the blank. The experiment was run in triplicates. The percentage inhibition of albumin denaturation was calculated by using the following equation.

(3) $$\begin{array}{c} \text{Inhibition }\left(\%\right)=\left[\frac{A_{\text{control}}–A_{\text{sample}}}{A_{\text{control}}}\right]\times 100, \end{array}$$

where *A*_control_ represents the absorbance of control and *A*_sample_ denotes the absorbance of the sample having extract/ibuprofen.

#### 2.2.5. Egg Albumin Denaturation Inhibition

Egg albumin denaturation inhibition method was used to investigate the anti-inflammatory potential of 6-gingerol. The reaction mixture (5 ml) contained 200 *μ*l of hen's egg albumin (fresh), 2.8 ml of phosphate buffer (pH 6.4), and 2 ml of various concentrations (100-600 *μ*g/ml) of 6-gingerol or standard drug (diclofenac sodium 200 *μ*g/ml), and 2 ml of distilled water was used instead of extract or diclofenac to prepare the control. The reaction mixtures were incubated for 15 min at room temperature in a BOD incubator, and then the reaction mixtures were heated at 70°C for 5 min. After cooling, the absorbance of reaction mixtures was measured at 660 nm using a UV-visible spectrophotometer using the buffer as the blank. The inhibition percentage of egg albumin denaturation was calculated using the following equation.

(4) $$\begin{array}{c} \text{Percentage inhibition of albumin denaturation }\left(\%\right)=\left[\frac{{\text{A}}_{\text{control}}–{\text{A}}_{\text{sample}}}{{\text{A}}_{\text{control}}}\right]\times 100, \end{array}$$

where *A*_control_ is the absorbance of control and *A*_sample_ is the absorbance in the presence of extract/diclofenac sodium.

#### 2.2.6. Evaluation of *In Vitro* Anti-Inflammatory Activity by Antiproteinase Action

The antiproteinase action of 6-gingerol was determined by the modified method of Sakat et al. [24]. For this purpose, 0.06 mg trypsin, 1 ml 20 mM Tris HCl buffer (pH 7.4), and 1 ml 6-gingerol of varying concentrations (100-600 *μ*g/ml) or diclofenac sodium (100 and 200 *μ*g/ml) in 2 ml reaction mixture were first incubated at 37°C for 5 minutes. After completion of incubation, 1 ml of 0.8% (*w*/*v*) casein was added. The mixture was incubated for an additional 20 minutes. After 20 minutes, the reaction was arrested by the addition of 2 ml of 70% perchloric acid, and the cloudy suspension was centrifuged for 5 minutes at 2500 rpm. The absorbance of the supernatant was recorded at 210 nm using a buffer as the blank. All the tests were run in triplicates. The percentage inhibition of proteinase inhibitory activity was calculated.

(5) $$\begin{array}{c} \text{Percentage inhibition}=\left[\frac{A_{\text{control}}–A_{\text{sample}}}{A_{\text{control}}}\right]\times 100, \end{array}$$

where *A*_control_ is the absorbance of the control and *A*_sample_ is the absorbance in the presence of 6-gingerol/diclofenac sodium.

### 2.3. *In Vivo* Study

#### 2.3.1. Animals

Adult male albino rats (6–7 weeks old, 175–200 g) were obtained from the King Saud University, Riyadh, Saudi Arabia. The rats were properly housed in polypropylene cages at 24 ± 5°C and exposed to a 12 h light/dark cycle. The rats were maintained on standard pellet diet and tap water and were given proper human care. The rats were acclimatized under standard laboratory conditions for a period of one week before the commencement of the experimental procedure. Animal utilization protocols were performed in accordance with the guidelines provided by the College of Applied Medical Sciences, Qassim University animal care and ethical committee.

#### 2.3.2. Experimental Design

Thirty-two rats were arbitrarily grouped into four groups of eight rodents in each group (*n* = 8). Rats belonging to group I or the control group received normal saline solution frequently. Group II was considered to be the disease control group and received DEN at a dose of 50 mg/kg body weight which was given three times in a week as per the previous described method with little modification [25]. Animals in group III were orally administrated with 6-gingerol at a dose of 50 mg/kg body weight before oral administration of DEN. Rats belonging to group IV were treated with 6-gingerol (50 mg/kg body weight) alone orally three times in a week as per the previously mentioned methods with slight modifications [26]. After the accomplishment of treatment for 8 consecutive weeks, the rats were subjected to ether anaesthesia. The animals were sacrificed to obtain the liver and blood samples.

#### 2.3.3. Measurement of Liver Function Enzymes and Total Protein and Albumin

After blood collection, the serum was separated by centrifugation at 3000 × *g* for 15 min at room temperature. Specific biochemical parameters which have been known to be altered due to hepatotoxins were evaluated to investigate the hepatoprotective activity of 6-gingerol. In the serum, aspartate aminotransferase (AST), alanine aminotransferase (ALT), and alkaline phosphatase (ALP) were spectrophotometrically assayed utilizing a commercial kit according to the manufacturer's protocol.

The concentration of total protein and albumin was estimated in all treated groups according to the manufacturer's instructions, and the results were interpreted accordingly.

#### 2.3.4. Estimation of Serum Lipid Profile Parameters

Estimation of serum lipid profile parameters, triglyceride (TG) and total cholesterol (TC), was assayed by enzymatic colorimetric test according to the manufacturer's protocol.

#### 2.3.5. Estimation of Glutathione (GSH), Superoxide Dismutase (SOD), and Glutathione-S-Transferase (GST) Level

Liver tissues obtained from each group of rats were homogenized in ice-cold 10% trichloroacetic acid phosphate buffer saline. The homogenates were centrifuged at 15000 × *g* for 10 minutes at low temperature. The supernatants were collected for the measurement of antioxidant enzymes including glutathione level (GSH), superoxide dismutase (SOD), and glutathione-S-transferase (GST) activities. The antioxidant enzymes were measured by a colorimetric method using commercial kits (Abcam, UK) according to the manufacturer's instructions.

#### 2.3.6. Measurement of Total Antioxidant Capacity

The total antioxidant capacity of each sample was measured according to the manufacturer's protocols using a commercial kit (Abcam, UK). Total antioxidant capacity assay can be used to measure either the combination of both small molecule antioxidants and proteins, or small molecule antioxidants only in the presence of a protein mask. In brief, both small molecules and protein antioxidants reduce Cu^2+^ ion to Cu^+^. However, the presence of the protein mask prevents reduction of Cu^2+^ by proteins. Thus, the protein mask allows the assay of small molecule antioxidant molecules only. The reduced Cu^+^ ion chelates with a colorimetric probe and gives a broad absorbance peak around 570 nm which is proportional to the total antioxidant capacity of a sample.

#### 2.3.7. Lipid Peroxidation Measurement

Lipid peroxidation was measured in liver tissue using a lipid hydroperoxide assay. Briefly, in the lipid hydroperoxide assay, lipid hydroperoxide was extracted from each experimental group sample as per the manufacturer's instruction. The chromogenic reaction was carried out at room temperature, the absorbance of each sample well was checked at 530 nm, the degree of lipid peroxidation was measured as thiobarbituric acid reactive substances (TBARS), and the result was expressed as *μ*mol of MDA equivalents per mg of protein.

#### 2.3.8. Assay of C-Reactive Protein (CRP), TNF-Alpha, IL-6, and ICAM1

An enzyme-linked immunosorbent assay (ELISA) of C-reactive protein, TNF-alpha, IL-6, and ICAM1 kit (Abcam, United Kingdom) was used to measure the concentration of all inflammatory markers according to the manufacturer's protocol.

#### 2.3.9. Histopathological Observations

The liver tissues were collected and fixed in 10% neutral buffered formalin, dehydrated in graded alcohol, cleared in xylene, embedded in paraffin wax, and sectioned to a thickness of 5 *μ*m. The sections were stained with haematoxylin and eosin (H&E staining) for histopathological studies. Finally, H&E-stained liver sections were examined using a Leica DM5000B microscope (Leica, Heidelberg, Germany), and subsequently, photographs were captured. The results were interpreted and scored as 0: no injury; 1: mild injury; 2: moderate injury; and 3: severe injury.

#### 2.3.10. Measurement of Collagen Fibers by Sirius Red Staining

Sirius red staining techniques were used to assess the fibrosis of the liver using Picro Sirius Red Kit (Abcam, UK).

#### 2.3.11. Masson Trichrome Staining

Masson trichrome staining was used to assess the collagen fiber of the liver using a Masson Trichrome Staining Kit (Abcam, UK). Concisely, sections were deparaffinised, and preheated Bouin's fluid had been added for one hour convoyed by a 10-minute cooling period. The slides were washed in tap water till the section became completely clean. The slides were stained with working Weigert's iron haematoxylin for five minutes. Biebrich Scarlet-Acid Fuchsin Solution was applied to the slides for 15 minutes. The differentiation in phosphomolybdic/phosphotungstic acid solution was performed for 10-15 minutes. Without rinsing, aniline blue solution was applied to the slides for 5-10 minutes. Acetic acid solution (1%) was applied to the slides. Dehydration was performed very quickly in 2 changes of 95% ethyl alcohol, followed by 2 changes of absolute alcohol, and the slides were cleared via xylene and mounting was done.

#### 2.3.12. Liver Ultrastructural Examination

Ultrastructural changes in the liver tissues were examined by electron microscope [27]. Concisely, liver tissues were collected and were immediately fixed in 2.5% glutaraldehyde buffered to pH 7.2 with phosphate buffer, and the tissues were postfixed in 1% osmic acid for 70 min. Subsequently, the samples were dehydrated by graded alcohol and were embedded in epoxy resin. Ultrathin sections were prepared using an Ultramicrotome (Leica UCT, Germany). These ultrathin sections were stained with 2% uranyl acetate and lead citrate. The ultramicrostructures of hepatocytes were examined under a transmission electron microscope (TEM). Digital images were captured with an integrated CCD camera.

#### 2.3.13. Expressional Evaluation of TNF-Alpha Protein

Tumor Necrosis Factor alpha (TNF-*α*) is an important cytokine and inflammatory mediator produced during inflammation. The expression pattern of TNF-*α* was evaluated through immune-histochemical staining as per the previously described method with some modifications [28, 29]. Concisely, formalin-fixed paraffin-embedded tissue blocks were cut into 5 *μ*m thick serial sections. The sections were deparaffinised, rehydrated, and rinsed in phosphate buffer saline. Antigen retrieval was made in a citrate buffer (pH 6.0) using the microwave method followed by incubation in 3% H_2_O_2_ for 20 minutes to block endogenous peroxidase activity. The sections were then incubated in a blocking agent for 1 hour at room temperature, followed by incubation with TNF-alpha primary antibodies overnight at 4°C. This step was followed by using secondary biotinylated antibody for 1 hour. Sections were washed in phosphate buffer saline and then incubated with streptavidin peroxidase for 45 minutes. Then, diaminobenzidine (DAB) and chromogen were used, sections were counterstained with haematoxylin, and the results were interpreted under a light microscope.

### 2.4. Statistical Analysis

All numerical data were interpreted in terms of mean ± SD expressed, and all comparisons were made by one-way analysis of variance (ANOVA) test. Statistical analysis was done by utilizing SPSS software. *p* < 0.05 was considered to be statistically significant.

## 3. Results

### 3.1. DPPH Radical Scavenging Assay

**Figure 1:**
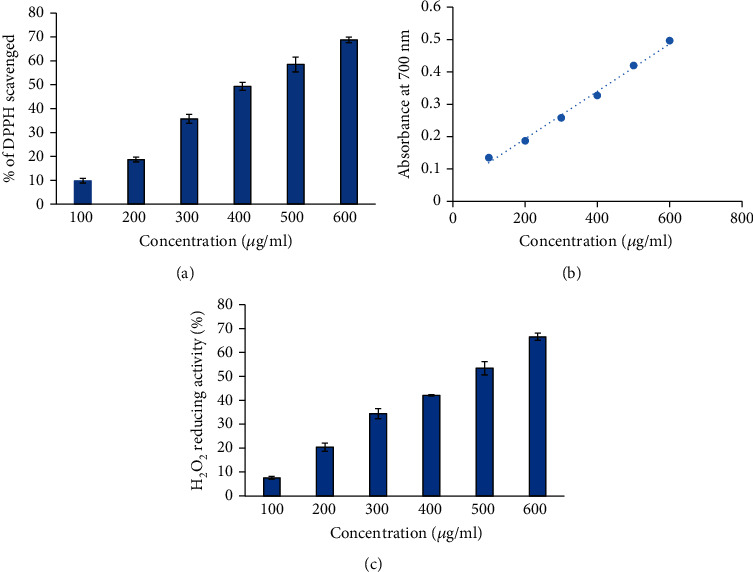
(a) Percent free radical scavenging activity of 6-gingerol. The bars show percent free radical activity vs. concentration (*μ*g/ml) of 6-gingerol (*p* < 0.05). (b) Reducing power of 6-gingerol in terms of absorbance at 700 nm. *x*-axis indicates the various concentrations of 6-gingerol. *y*-axis shows the corresponding absorbance at 700 nm. (c) H_2_O_2_ reducing activity (%) of 6-gingerol. Samples 1 to 6 correspond to various concentrations of 6-gingerol (100–600 *μ*g/ml). The results are presented as means ± SEM (n = 3, *p* < 0.05).

The DPPH assay is a significant method used to determine the antioxidant activity of various substances. In this assay, the antioxidant molecule provides an electron to DPPH and it gets reduced and generates yellow-colored diphenyl-picryl hydrazine. The reduction of DPPH by antioxidants can be measured by recording the decrease in absorbance at 517 nm. Our findings show that 6-gingerol has an excellent free radical scavenging activity, and this activity was found to be highest at 600 *μ*g/ml. The strong free radical scavenging activity of 6-gingerol indicates its potent antioxidant nature. The results of DPPH free radical scavenging activity assay are shown in Figure 1(a).

### 3.2. Ferric Reducing Antioxidant Power (FRAP) Assay

The antioxidant capacity of 6-gingerol was evaluated by FRAP assay, and ascorbic acid was used as the standard. Total antioxidant capacity is linked with reduction of Fe^+3^ to Fe^+2^ by 6-gingerol. The ascorbic acid solution (50-250 *μ*g/ml) conformed to Beer's Law at 700 nm with a regression coefficient (*R*^2^) = 0.9938. The plot has a slope (*m*) = 0.0034 and intercept = 0.0258. The equation of standard curve is *y* = 0.0034*x* + 0.0258. FRAP value of 6-gingerol was found to be 35.75 ± 0.0769 *μ*g ascorbic acid/100 mg dry weight of extract. The reducing power of 6-gingerol in terms of absorbance was found to be increased in a concentration-dependent manner (Figure 1(b)). Values are in mean ± SEM.

### 3.3. Hydrogen Peroxide (H_2_O_2_) Reducing Activity

Reduction of H_2_O_2_ for various dilutions of 6-gingerol may be due to its electron-donating ability and was assessed according to a previously described method [22]. H_2_O_2_ reducing activity of 6-gingerol was found to increase in a concentration-dependent manner (Figure 1(c)). The maximum percentage of H_2_O_2_ reducing activity was exhibited by 600 *μ*g/ml of 6-gingerol (Figure 1(c)). This reducing activity of 6-gingerol is very crucial because the lethal hydroxyl radicals are produced by disintegration of H_2_O_2_. Hydroxyl radicals are harmful because they are cytotoxic in nature [28].

### 3.4. Albumin Denaturation Inhibition Activity

**Figure 2:**
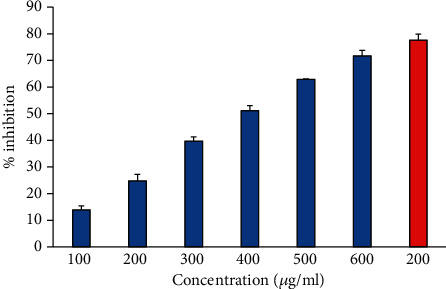
The percentage inhibition of heat-induced denaturation. Columns (from 1 to 6) at *x*-axis represent concentration 100-600 *μ*g/ml of 6-gingerol. Red colored column shows 200 *μ*g/ml of ibuprofen. The results are presented as means ± SEM (*n* = 3, *p* < 0.05).

During inflammation and arthritis, proteins are significantly denatured. Thus, a protein denaturation inhibition assay could be very useful for investigating the anti-inflammatory potential of any natural product. It is considered that the agents having protective potential against protein denaturation would be used in anti-inflammatory drug development [29] Our finding showed that heat-induced albumin denaturation was inhibited by 6-gingerol. Moreover, the percent inhibition of albumin denaturation was found to be increased in a concentration-dependent manner (Figure 2). Ibuprofen was used as a reference anti-inflammatory drug that showed the maximum inhibition, 77.59 ± 2.31% at the concentration of 200 *μ*g/ml.

### 3.5. Inhibition of Egg Albumin Denaturation

**Figure 3:**
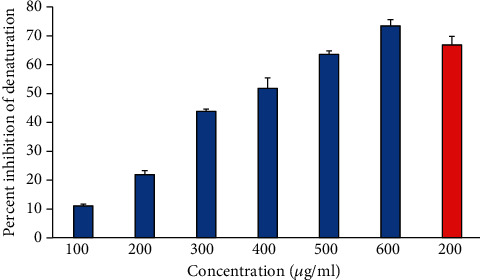
Percentage protection of heat-induced denaturation of egg albumin. Blue columns (1-6) at *x*-axis represent concentration 100-600 *μ*g/ml of gingerol, and red column shows concentration 200 *μ*g/ml of diclofenac. The results are presented as means ± SEM (*n* = 3, *p* < 0.05).

The antiarthritic effect of 6-gingerol was investigated by using the inhibition of egg albumin denaturation (Figure 3). 6-Gingerol inhibited egg albumin denaturation in a dose-dependent manner (100-600 *μ*g/ml). The current study indicates that 6-gingerol is highly effective against heat-induced denaturation of egg albumin, and the denaturation of egg albumin decreases with an increase in the concentration of 6-gingerol. Diclofenac, a standard drug, showed the maximum inhibition (66.786 ± 2.95%) at a concentration of 200 *μ*g/ml.

### 3.6. Antiproteinase Activity

**Figure 4:**
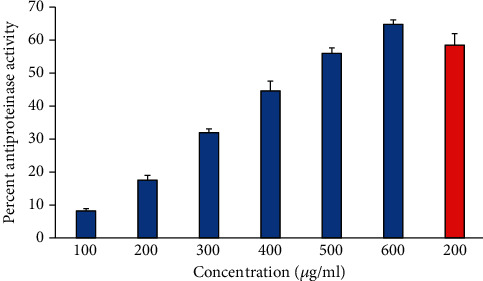
Antiproteinase activity of 6-gingerol. Samples 1-6 represent various concentrations (100-600 *μ*g/ml) of 6-gingerol. Last two samples 7 and 8 contained 100 and 200 *μ*g/ml of diclofenac sodium.

Proteinases are enzymes that are known to be implicated in arthritic reactions. Antiproteinase activity of natural products can be linked with controlling inflammatory disorders because of its potential to offer protection from proteinase-induced tissue damage. The data suggest that 6-gingerol possesses a considerable antiproteinase activity, and the antiproteinase activity of 6-gingerol was found to increase in a concentration-dependent manner, and 6-gingerol at 600 *μ*g/ml showed maximum antiproteinase activity. However, reference drug “diclofenac sodium” exhibited maximum inhibition at 200 *μ*g/ml (Figure 4).

### 3.7. Effect of 6-Gingerol on Serum AST, ALT, and ALP and Total Protein and Albumin

**Figure 5:**
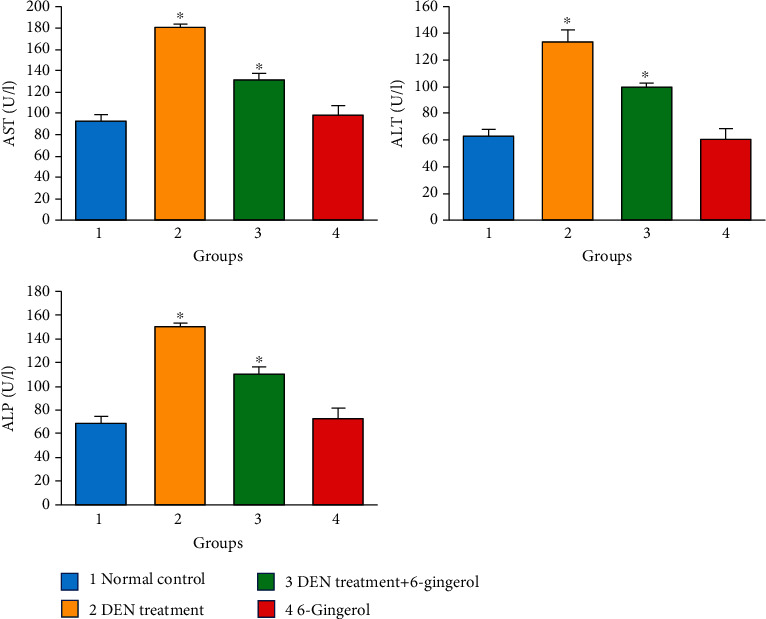
The role of 6-gingerol on liver function enzymes. ALT, ALP, and AST enzyme levels were considerably higher in the group of DEN-induced rats than in the control group. However, the group that got both DEN and 6-gingerol demonstrated substantially lower ALP, ALT, and AST levels than the DEN-induced group only. Statistical significances are compared between the normal control vs. DEN-induced groups only (*p* < 0.01), and DEN-induced versus both DEN- and 6-gingerol-treated groups (*p* < 0.05).

**Figure 6:**
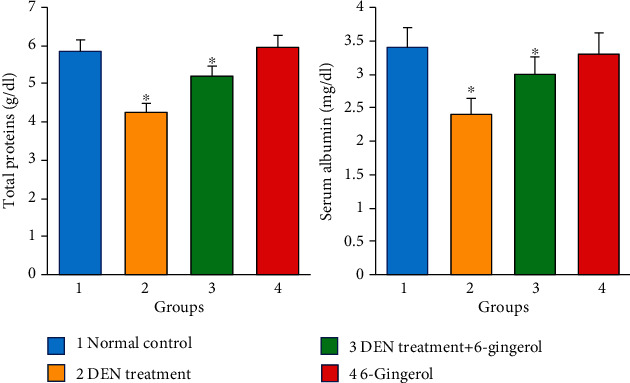
Significant reductions in the concentration of total protein and albumin were seen in the DEN-treated group, and pretreatment with 6-gingerol significantly increased the concentrations of such proteins (*p* < 0.05).

Hepatotoxicity and liver damage are commonly linked with marked elevation in the levels of ALT, AST, and ALP enzymes. In our study, DEN administration rats developed severe hepatotoxicity as reflected by a significant increase in AST, ALT, and ALP levels (*p* < 0.05) as compared to the control group. The level of these enzymes significantly decreased (*p* < 0.05) towards normal levels in rats that received 6-gingerol plus DEN when compared to the DEN-treated group (Figure 5). AST, ALT, and ALP levels were found to be in the normal range in rats that received 6-gingerol only. Moreover, significant reduction in the concentration of total protein and albumin was seen in the DEN-treated group. The pretreatment with 6-gingerol significantly increased the concentrations of the proteins in the serum (*p* < 0.05) (Figure 6).

### 3.8. Effect of 6-Gingerol on the Lipid Profile of Rats Exposed to DEN

**Figure 7:**
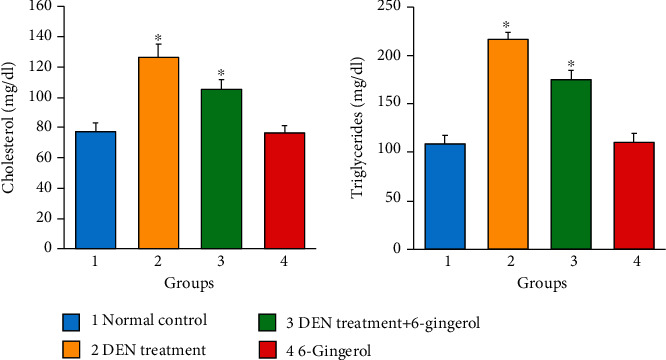
DEN induction caused significant increase in serum lipids including cholesterol and triglycerides as compared to the control. The coadministration of 6-gingerol with DEN showed a significant change as compared to the DEN-treated group (*p* < 0.05).

Administration of DEN caused significant increase in serum lipids including cholesterol and TGs in rats as compared to the control. The coadministration of 6-gingerol with DEN showed significant changes, and the difference was statistically significant as compared to the DEN-treated group (*p* < 0.05) (Figure 7).

### 3.9. Effect of 6-Gingerol on Antioxidant Enzyme Status in Rats Exposed to DEN

**Figure 8:**
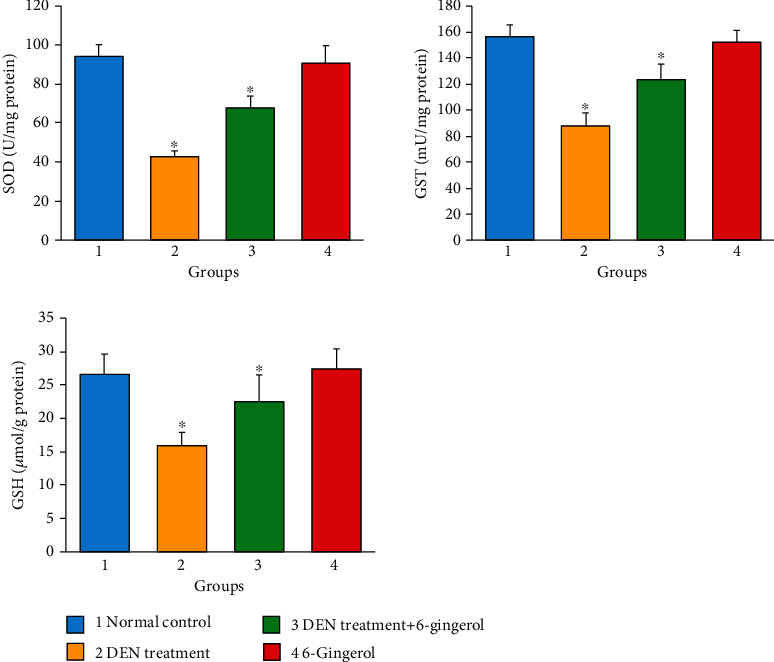
Glutathione (GSH), superoxide dismutase (SOD), and glutathione-S-transferase (GST) level in the different treatment groups. DEN-treated groups showed a decreased level of GSH, SOD, and GST enzymes as compared to the control group (*p* < 0.05). Treatment of 6-gingerol and DEN increased the levels of these enzymes considerably as compared to the DEN-treated group (*p* < 0.05).

Our findings show that there was a significant decrease in the glutathione level (GSH), superoxide dismutase (SOD), and glutathione-S-transferase (GST) concentrations due to DEN administration as compared to the control group. Treatment with 6-gingerol prior to DEN administration significantly increased the hepatic GSH, SOD, and GST activities as compared to the DEN-treated group (Figure 8). The difference in antioxidant enzyme level in the DEN-treated group and the group that received 6-gingerol with DEN was statistically significant (*p* < 0.05).

### 3.10. 6-Gingerol Inhibits Lipid Peroxidation in DEN-Induced Liver Toxicity

**Figure 9:**
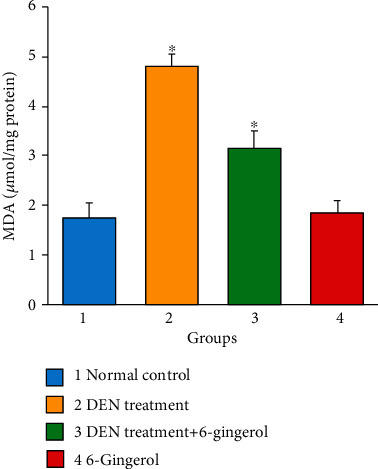
Effects of DEN exposure on hepatic lipid peroxidation. The formation of malondialdehyde (MDA) was analyzed in control, disease control (DEN treated), DEN+6-gingerol treatment, and only 6-gingerol treatment groups. Each bar represents the mean value of experiments performed in triplicate ± SEM (*n* = 8, each group). ^∗^*p* < 0.05 compared with the control group.

The high levels of reactive oxygen species (ROS) cause membrane lipid peroxidation and the production of linked by-products like malondialdehyde (MDA) and 4HNE [30]. As a product of lipid peroxidation, the malondialdehyde level can reflect the liver lipid peroxidation level [31]. The DEN induction caused an increase in malondialdehyde (MDA) production due to an increase in membrane lipid peroxidation. DEN treatment caused significant increases in MDA levels as compared to the control group (*p* < 0.05). 6-Gingerol treatment resulted in a significant reduction in the elevated MDA levels compared with the DEN-treated group (*p* < 0.05) (Figure 9).

### 3.11. Effect of 6-Gingerol on Inflammatory Marker Status in Rats Exposed to DEN

**Figure 10:**
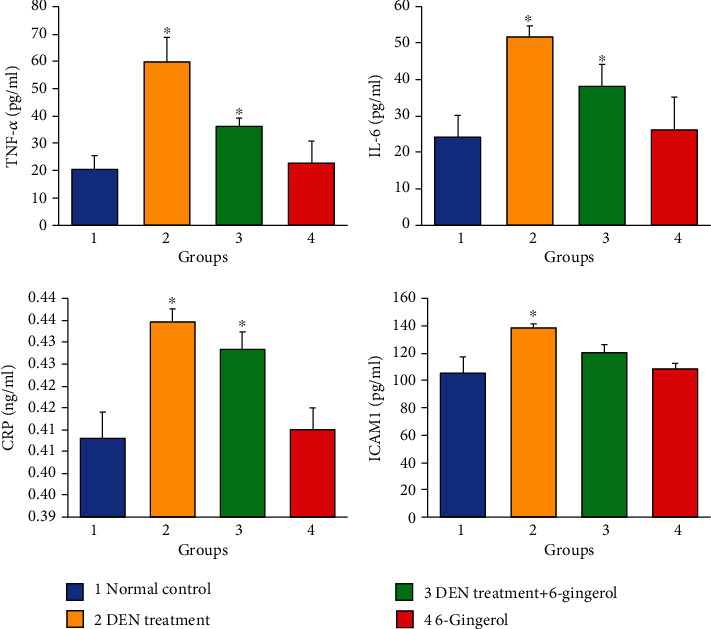
The effect of 6-gingerol on inflammatory markers. The anti-inflammatory markers include TNF-*α*, IL-6, CRP, and ICAM1. In rodents, which received DEN, the levels of TNF-*α*, IL-6, CRP, and ICAM1 were found to be significantly increased as compared with the control group (*p* < 0.05). However, the levels of these inflammatory markers were significantly decreased in rats administrated with both DEN and 6-gingerol (*p* < 0.05).

The inflammatory markers including TNF-*α*, IL-6, ICAM1, and CRP were found to be significantly increased in DEN-induced hepatotoxic rats as compared to the control group (*p* < 0.05) (Figure 10). The treatment with 6-gingerol significantly decreased the inflammatory marker levels (*p* < 0.05).

### 3.12. Effect of 6-Gingerol on Liver Architecture

**Figure 11:**
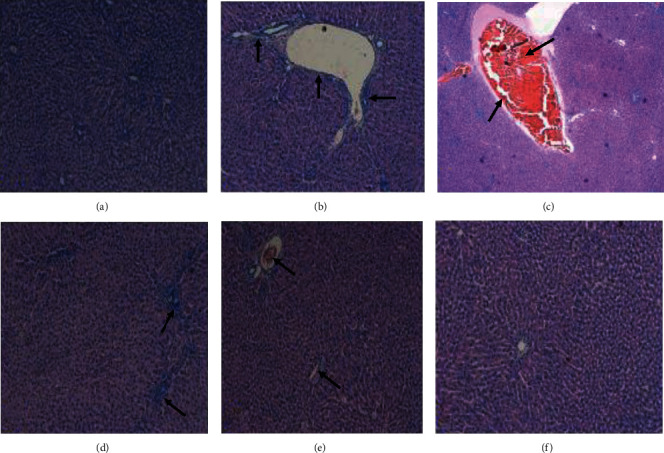
Histopathological analysis. Liver tissues showing (a) the normal architecture of hepatocytes in liver sections of control group rats. DEN-only treated tissues showing various types of alterations (arrows shown) such as blood vessel dilation, congestion, and increased inflammatory cells (b–d). 6-Gingerol plus DEN-treated tissue showed that liver tissue alteration was significantly less as compared to the DEN-treated group (arrows shown) (e). 6-Gingerol-only treated group shows normal hepatocyte architecture (f).

Liver tissues of the control group rats showed normal architecture of hepatocytes such as clear cellular boundary, rounded and centrally located nuclear membrane, central lobular vein, and sinusoids with no dilation or congestion (Figure 11). The liver sections of rats treated with DEN showed severe injury characterized by infiltration of inflammatory cells, congestion, blood vessel dilation, and edema as compared with those of the control group (Figure 11). In addition, hepatic damage was found to be reduced in rats having coadministration of 6-gingerol with DEN treatment, and the histological changes like infiltration of inflammatory cells, blood vessel dilation, and edema were decreased (Figure 11).

### 3.13. Measurement of Fibers by Sirius Red Staining

**Figure 12:**
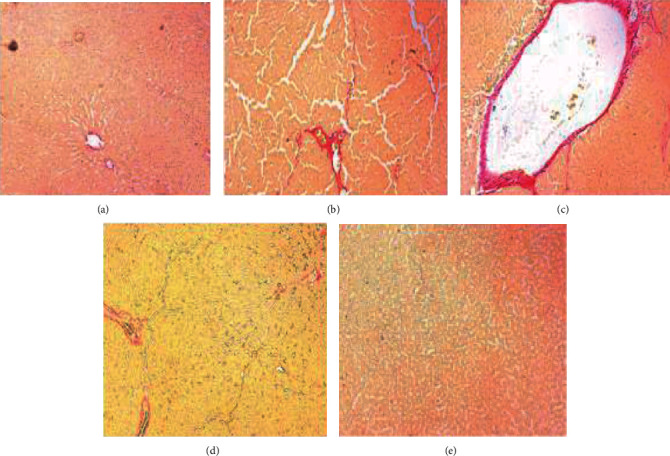
Sirius red staining analysis. Liver tissues showing the normal architecture of hepatocytes (a). DEN-only treated tissues showing significant fiber (b, c). 6-Gingerol plus DEN-treated tissue showed much lower collagen fiber as compared to the DEN-treated group (d). 6-Gingerol-only treated group showed normal hepatocytes and less fibers (e).

The fiber deposition was found to be almost 20% of the Sirius red positive in the liver tissue of rats exposed to DEN alone as compared to the control group which showed less than 5% of Sirius red positive. Besides, collagen fibers were considerably lower in 6-gingerol-treated rats (almost 10%) than those rats exposed to DEN alone. Based in these findings, it is revealed that 6-gingerol efficiently decreased the fiber deposition in the liver tissues (Figure 12).

### 3.14. Evaluation of Collagen Fiber by Masson Trichrome Staining.

**Figure 13:**
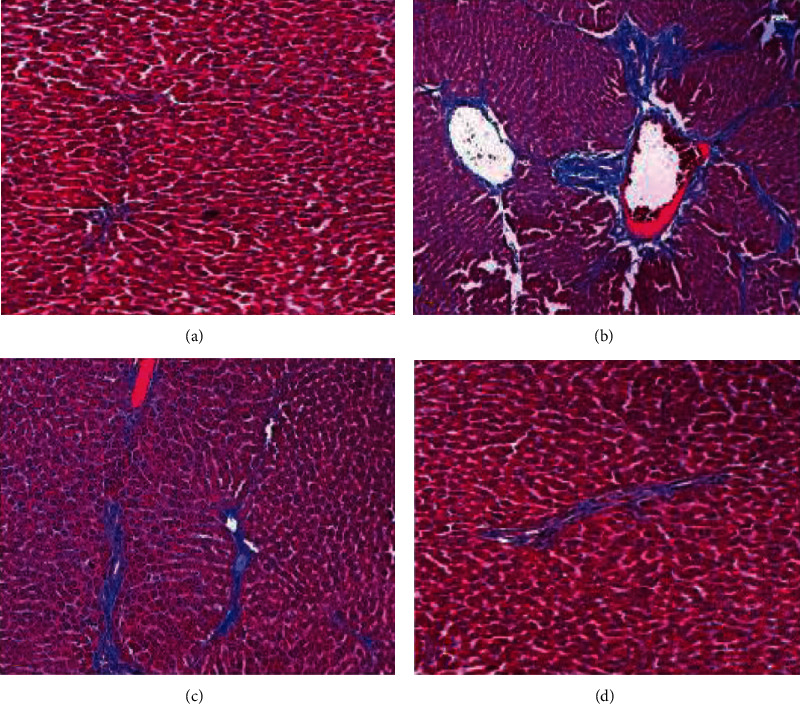
Masson trichrome staining analysis. Liver tissues showing the normal architecture of hepatocytes (a). DEN-only treated tissues showing significant collagen fiber (b). 6-Gingerol plus DEN-treated tissue showed much lower collagen fiber as compared to the DEN-treated group (c). 6-Gingerol-only treated group showed normal hepatocytes and less fibers (d).

As shown by Masson trichrome staining, there were more collagen fibers in the DEN-treated group compared with the normal group. The collagen deposition was significantly decreased 6-gingerol plus DEN-treated tissue compared with the DEN-treated tissue (Figure 13). The 6-gingerol-only treated group showed normal hepatocytes and less collagen fibers.

### 3.15. Ultrastructural Examination

**Figure 14:**
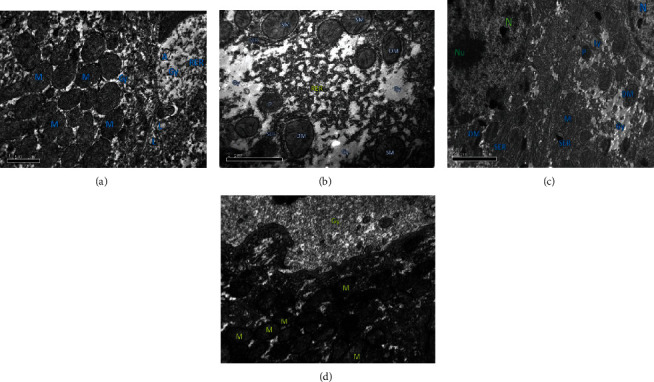
Transmission electron micrographs represent the ultrastructural features of liver tissues of rats from each experimental group. (a) Control group showed numerous mitochondria (M)—mitochondria were spherical and round-shaped, depending on the location within the cell; clumps of glycogen surrounded (Gy), adipocytes (A), lysosome (L), and dense granulated cytoplasm were seen in the control group. (b, c) DEN-treated group tissues showing few mitochondria (M), and some of them are losing their shape (DM) and swollen mitochondria (SM), presence of glycogen clump (Gy), and broken SER. (c) Nucleus (N) with initial stage of chromatin disintegration (Nc) and few mitochondria (M), and some of them are losing their shape (DM), presence of glycogen clump (Gy), well-developed SER-ER tubule, few lysosomes (Ly), and presence of few peroxisomes (P). (d) DEN plus 6-gingerol-treated group tissues showed the presence of numerous small mitochondria (M); most of the mitochondria are spherical or round normal shaped, with dense granulated cytoplasm (C), presence of glycogen clump (Gy), and presence of lysosome (Ly).

To further investigate the hepatoprotective effects of 6-gingerol, ultrastructural examination of liver tissue was conducted by transmission electron microscopy (TEM) (Figure 14). In control group rats, numerous mitochondria (M) were found to be spherical and round-shaped, depending on the location within the cell. Besides, clumps of glycogen surrounded (Gy), adipocytes (A), lysosomes (L), and dense granulated cytoplasm were noticed in the control group rats. In the DEN-treated group rats, tissues showed few mitochondria (M), some of them were losing their shape (DM), and some were swollen mitochondria (SM). Further, the presence of a glycogen clump (Gy) and broken SER was also noticed in liver tissues of DEN-treated rats. The tissues of rats treated with both DEN and 6-gingerol also exhibited some alterations such as the nucleus (N) with the initial stage of chromatin disintegration (Nc) and few mitochondria (M), and some of them were shown to lose their shape (DM). Other features in tissues of this group included the presence of glycogen clump (Gy), well-developed SER-ER tubules, few lysosomes (Ly), and the presence of few peroxisomes (P). However, the severity of changes in this group was lesser as compared to that in the DEN-treated group (Figure 14).

### 3.16. Expression of TNF-*α* Protein with Immunohistochemistry

**Figure 15:**
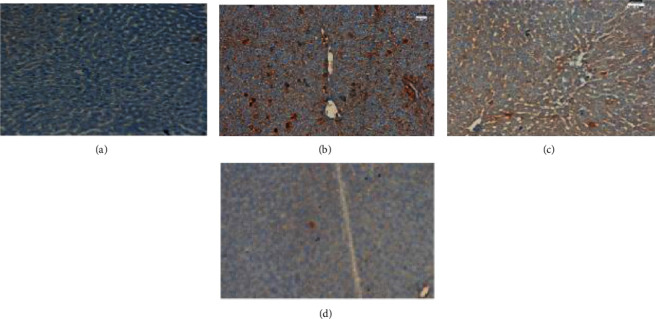
Immunohistochemical analysis of TNF-*α*. TNF-*α* protein expression was evaluated in different treatment groups (a–d). (a) The control group did not show any expression of TNF-*α* protein; (b) the DEN-treated group showed high expression of TNF-*α* protein; TNF-*α* expression was also noted in (c) rats treated with both DEN and 6-gingerol; and (d) 6-gingerol-only treated group did not shown any expression.

TNF-*α* protein expression was analyzed in experimental groups through immunohistochemical staining (Figure 15). A total of five fields from each section were selected, 100 cells from each area were counted, and the mean percentage positivity was calculated. The expression of TNF-*α* was considered positive if more than 5% of cells showed positivity, and less than 5% positivity was taken as the negative case. The percentage of IHC staining intensity of cytoplasmic TNF-*α* was considered mild when positive staining is <20% and moderate when expression of TNF-*α* was 25 to 50%, and when positive staining was more than 50%, it was considered as a strong positive. The significantly increased expression of the TNF-*α* protein was noticed in rats treated with DEN only. Moreover, TNF-*α* protein expression was significantly decreased in rats administrated with both 6-gingerol and DEN. However, the TNF-*α* expression in the treatment group receiving 6-gingerol alone was similar to the control group. The expression pattern of TNF-*α* among different treatment groups was statistically significant.

## 4. Discussion

The present study investigated the *in vitro* antioxidant and anti-inflammatory activities and potential hepatoprotective activity of 6-gingerol through *in vivo* studies. Several factors which are involved in the pathogenesis of liver include oxidative stress and lipid peroxidation [32, 33]. Diethylnitrosamine (DEN) is a well-known hepatocarcinogen, and it induces liver injury and is usually used to induce liver cancer in experimental animal models [7, 34–36]. It has been reported that the prolonged oral administration or intraperitoneal injection of DEN causes liver pathogenesis.

The elevated levels of liver function enzymes in the serum are associated with hepatocellular damage because damage in the liver cell plasma membrane results in the release of these enzymes into circulation [37–40]. ALT and AST are basically found in the hepatic cells, and their leakage is correlated to cellular damage. Therefore, serum levels of ALT and AST can be considered as the most sensitive indicators of hepatocyte injury [41, 42]. Our results demonstrated that DEN administration (50 mg/kg b.w.) developed severe liver injury in rats that was characterized by a significant increase in serum enzyme activity of AST, ALT, and ALP. The results had showed that administration of 6-gingerol considerably inhibited DEN-induced elevation of serum ALT, ALP, and AST levels, indicating that 6-gingerol protected the liver from the damaging effects of DEN hepatotoxin, which was well supported by our histological findings.

Further, our results were in accordance with previous findings of some investigators who reported that treatment of 6-gingerol and silymarin had significant hepatoprotective effect against acetaminophen-induced hepatotoxicity as indicated by the decrease in serum levels of liver marker enzymes including AST, ALT, and ALP [43]. Besides, another previous study has confirmed the hepatoprotective activity of ginger. An administration of ginger has exhibited the decrease in the elevated serum level of AST, ALT, and ALP [44]. In addition, ginger treatment prior to acetaminophen displayed a significant hepatoprotective effect because of the decrease in the concentration of hepatic marker enzymes and total bilirubin in plasma [45].

Oxidative stress is explained as an imbalance between production and removal of ROS due to the decrease in the antioxidant defense mechanisms [46]. It is very evident that free radicals adversely affect various types of biomolecules and can cause lipid peroxidation, thereby damaging several organelles. If endogenous antioxidant enzymes become inactivated, the oxidative stress becomes exacerbated [47]. ROS have also been reported to be produced by metabolism of hepatotoxic substances. DEN interacts with macromolecules and leads to lipid peroxidation of the cell membrane.

Endogenous antioxidative enzymes including superoxide dismutase (SOD), catalase (CAT), and glutathione peroxidase (GPx) protect the liver against oxidative damage [48] because they convert ROS into stable molecules such as water and O_2_ [49]. SOD is an important part of the antioxidant enzyme that encounters the harmful effects of ROS [50, 51]. Ginger and its active compound 6-gingerol have been documented to possess significant antioxidant property, and such activities have been reported to play a considerable role in the activation of antioxidation protective cascades. Further, this activity is attributed to the reduction of the liver damage through lowering of hepatic marker enzymes. Our findings from the DPPH assay confirmed the strong antioxidant nature of 6-gingerol. Thus, our result indicates that high antioxidant capacities of 6-gingerol might contribute to its therapeutic potential since it shows that its free radical scavenging ability may offer protection from oxidative damage caused by free ROS. The current study revealed that DEN administration caused a significant decrease in concentration of antioxidant enzymes including GSH, SOD, and GST in the livers of rats as compared to the control group. On the other hand, pretreatment of the rats with 6-gingerol before DEN administration significantly increased the activities of hepatic GSH, SOD, and GST activities. Thus, these observations provided evidences that 6-gingerol has the potential to protect liver damage induced by oxidative stress through enhancing activities of various antioxidant enzymes.

Recent studies have revealed that inflammation is generally implicated in the liver diseases ranging from the initial to the late stage [52] and is a common pathophysiological response to liver injury. Remarkably, the overexpression of proinflammatory cytokines results in the elevation of ROS and, hence, oxidative stress, causing cell damage [53, 54]. Keeping in mind this fact, it can be suggested that any antioxidant compound with anti-inflammatory activity can be used as a drug therapy in the management of liver damage. In addition, proinflammatory cytokines are documented to be produced in a large amount which has been considered to be an important hallmark of the pathogenic conditions including hepatic inflammation and injury. Carcinogens and toxins have been reported to cause tissue injury that significantly increases the levels of proinflammatory cytokines. Various types of proinflammatory markers like TNF-alpha and IL-6 have been found to contribute to the pathogenesis of liver diseases. Interleukin 6 (IL-6), quickly produced in response to different infections and tissue injuries, attributes response to host defense through the stimulation of acute phase responses, hematopoiesis, and immune reactions [55], and its overexpression has been noticed in liver diseases. After liver injury, nuclear factor-kappa B (NF-*κ*B) is activated that results in the production of various inflammatory factors including IL-6 and TNF-*α* [56]. IL-6 and TNF-*α* are considered as proinflammatory markers and are useful for the identification of low-grade inflammation associated with visceral adiposity [57].

Natural products have been already proven to inhibit the inflammatory marker level and inhibit the pathogenesis [58–61]. In the present study, DEN was found to cause significant elevation in the levels of inflammatory markers in DEN-induced rats as compared to the control group. The treatment with 6-gingerol was found to significantly reduce the levels of inflammatory markers. Therefore, our results suggested that 6-gingerol might inhibit DEN-induced inflammation through inhibition of inflammatory process/proinflammatory markers. There are many studies that are in accordance with our observations. A pioneer study reported that diethylnitrosamine induced elevation in liver function enzymes with noteworthy increase in oxidation; also, inflammation biomarkers and DENA caused degenerative changes in hepatocytes and inflammatory foci, whereas ginger extract pretreatment showed a role in the improvement of liver function and restored normal GSH with significant mitigation of oxidative stress and inflammatory biomarkers compared to the DENA-treated group [62].

A previous study had exhibited that IL-1*β*-induced inflammation and oxidative stress becomes attenuated by *S*-[6]-gingerol in HuH7 cells. This observation was supported by a decrease in mRNA levels of inflammatory factors such as IL-6 and IL-8. Further, S-[6]-gingerol was found to reduce both IL-1*β*-induced COX2 upregulation and NF-*κ*B activity [63]. In another study, 6-gingerol was shown to forestall the increase in inflammatory markers such as myeloperoxidase, NO, and TNF-alpha in the brain, ovaries, and uterus of rats treated with chlorpyrifos. Besides, 6-gingerol was noticed to restrain the LPS-induced increment in levels of GFAP and TNF-*α* in the rat brain [64].

In the current study, DEN-induced liver injury is confirmed by haemorrhages, infiltration of inflammatory cells, congestion, blood vessel dilation, and edema. In the meantime, coadministration of 6-gingerol significantly reduced the DEN-induced hepatic damage, and the histopathological patterns of liver injury were significantly less than those of rats belonging to the DEN treatment group. Henceforth, electron microscopy was used to observe the ultrastructure of hepatocytes. It was observed that alteration in cellular structures was more pronounced in the DEN-treated group. In contrast, treatment with 6-gingerol showed improved hepatocellular architecture. This supports our biochemical observations indicating the protective role of 6-gingerol against DEN-induced liver damage. Our findings are again supported by previous investigations regarding the protection of natural products against DEN-induced liver injury. It has been seen that administration of *P. granatum* and *Vitis vinifera* extracts to rats provides protection from the hepatocellular injury induced by DEN [65].

In the pathological process of liver injury, several inflammatory cytokines are meaningfully upregulated including TNF-*α*, which facilitate inflammation and repair in physiological conditions [66]. In the current study, it was noticed that the TNF-*α* expression was significantly high in the DEN-treated group, whereas the expression was notably decreased by the treatment of rats with 6-gingerol. Thus, our findings suggest that 6-gingerol exhibits hepatoprotective activity due to its anti-inflammation potential. These findings are consistent with the previous study on hepatoprotective activity of other medicinal plants. The expression of TNF-*α* was evaluated in the group receiving berberine alone and was found to be similar to the control. On contrary, a strong TNF-*α* expression was reported in rats receiving CCl_4_ only [67].

Our *in vivo* data has demonstrated that 6-gingerol extract possesses significant anti-inflammatory potential. Further, we confirmed the anti-inflammatory actions of 6-gingerol using an *in vitro* study. Thus, the results obtained from the antiproteinase activity assay are in accordance to our findings from *in vivo* experiments, and 6-gingerol has been exhibited to possess powerful anti-inflammatory action practically identical to the reference anti-inflammatory drug. Inhibition of proteinase action and inflammation may be contributed by its strong antioxidant potential. Thus, our data propose that significant antioxidant activity of 6-gingerol might be responsible for its anti-inflammatory activity, and administration of 6-gingerol can be suggested to contain some considerable protective effect against DEN-induced liver damage.

## 5. Conclusion

In conclusion, the findings of the present study demonstrate that DEN induces oxidative stress and hepatocellular changes in the liver, and 6-gingerol has a significant hepatoprotective effect against DEN-induced liver damage in rats through attenuation of oxidative stress and inflammation. Further, our results confirm that 6-gingerol reduces or prevents hepatic damage and enhances the recovery from liver injury induced by DEN. Based on the findings, it can be suggested that the hepatoprotective action of 6-gingerol might be mediated through its antioxidant nature and anti-inflammatory action. Thus, intake of 6-gingerol may have beneficial effects in treating liver diseases.

## Data Availability

The data used to support the findings of this study are included within the article.
